# Gambling problems among Lebanese adults: Arabic-Language version of the South Oaks Gambling Screen (SOGS) scale validation and correlates

**DOI:** 10.1186/s40359-022-00727-6

**Published:** 2022-02-01

**Authors:** Patrick Haddad, Roger Roukoz, Marwan Akel, Souheil Hallit

**Affiliations:** 1 Faculty of Medicine, Saint-Joseph University, Beirut, Lebanon; 2 School of Pharmacy, Lebanese International University, Beirut, Lebanon; 3 Faculty of Medicine and Medical Sciences, Holy Spirit University of Kaslik (USEK), Jounieh, Lebanon; 4 Research Department, Psychiatric Hospital of the Cross, Jal Eddib, Lebanon

**Keywords:** Gambling disorder, Pathological gambling, South Oaks Gambling Screen, SOGS, Lebanon, Depression, Alcohol use disorder

## Abstract

**Background:**

In the DSM-5, gambling disorder is recognized as the only behavioral addiction. The number of people with mental health disorders is increasing in Lebanon, especially since the economic crisis and the COVID-19 pandemic. The South Oaks Gambling Screen (SOGS), the most used tool in the world in terms of pathological gambling, has never been translated to Arabic, culturally adapted in a way that suits the Lebanese population and validated. This study’s objectives were to validate an Arabic-language version of the SOGS and assess factors associated with probable pathological gambling among Lebanese adults.

**Methods:**

This study was carried out between February and April 2021, during the lockdown period imposed by the Lebanese government. A total of 601 individuals participated in this study by filling the online questionnaire.

**Results:**

A factor analysis, using the principal component analysis, was performed on the SOGS scale items. The SOGS items were able to explain 73.35% of the variance, with an internal reliability of KR20 = 0.947 for the total scale. The results of the confirmatory factor analysis confirmed the results of the factor analysis. More problematic alcohol use (aOR = 1.17), and more depression (aOR = 1.13) were significantly associated with higher odds of probable pathological gambling, whereas females (aOR = 0.27) had significantly lower odds of probable pathological gambling compared to males.

**Conclusion:**

Our study validated an Arabic-language version of the South Oaks Gambling Screen (SOGS) for use in Lebanon, and showed some factors associated with probable pathological gambling (male gender, alcohol use disorder and depression). This reliable and valid version will hopefully contribute towards better screening for gambling disorder in Lebanon.

## Introduction

The term “addiction” is a broad term. It includes substance addictions, which are the most common addictions, including alcohol, drugs, tobacco, medication, and caffeine. But this term also encompasses behavioral addictions. In the DSM-5, the latest and fifth edition of the Diagnostic and Statistical Manual of Mental Disorders, “gambling disorder” is officially recognized as the only behavioral addiction [1]. In the DSM-IV, “pathological gambling” was not recognized as an addiction, but was classified as an impulse control disorder [2].

Gambling disorder is defined as a disordered gambling behavior that is persistent and recurrent. Its diagnosis is obtained by the DSM-5 criteria [1]: Problematic gambling behavior leading to clinically significant impairment or distress, that is not better explained by a manic episode. According to the ICD-11, gambling disorder is characterized by a pattern of persistent or recurrent gambling behavior, which may be online (i.e., over the internet) or offline, manifested by impaired control over gambling, increasing priority given to gambling to the extent that gambling takes precedence over other life interests and daily activities, and continuation or escalation of gambling despite the occurrence of negative consequences, that is of sufficient severity to result in significant impairment in functioning, over a period of at least 12 months [3].

Gambling disorder is often underdiagnosed. Nevertheless, neither the efficacy of screening for gambling disorder in the primary care setting, nor the optimal screening tool, has been established. The most used tool in the world in terms of pathological gambling is the South Oaks Gambling Screen (SOGS), developed by Henry Lesieur and Sheila Blume and published in 1987 in the American Journal of Psychiatry [4]. The SOGS format allows for many ways of administration, by experts, non-experts or by self-administration, which makes it more convenient and efficient than other scales, and explains why the SOGS quickly became the dominant instrument for measuring probable pathological gambling in research contexts [5].

Several studies have presented psychometric statistics for the SOGS [6–8], although there are relatively fewer for other scales like the Canadian Problem Gambling Index (CPGI): Given the relatively recent development of the CPGI, it is not surprising that there are fewer studies of its reliability and validity [9]. For this reason, and because of the lack of other validated instruments, the SOGS has enjoyed more extensive use than other scales, and is the most often used tool by Lebanese physicians to screen for gambling disorder in clinical settings. The SOGS is a lifetime-based measure, and uncovers individuals who are in remission and at risk for relapse. Therefore, the SOGS tends to over-estimate gambling problems when lifetime estimates are used and may not be necessarily the best indicator of the number of people who currently are experiencing gambling-related problems. One suggested solution is to change the time frame from lifetime to a more current period; researchers have used past 6 and 12 months’ time windows [10]. However, given the fact that there is a paucity of information on Lebanese gambling, a lifetime time frame is an important indicator of the potential burden on the Lebanese community and was used in our study, especially that the original SOGS is the screen most often used by Lebanese clinicians.

Other less used screening tools were developed; the Gamblers Anonymous Survey, which includes 20 questions, may be helpful in collecting clinical information and can help lead the patient to the Gamblers Anonymous program. Seven positive responses suggest the diagnosis of gambling disorder [11]. Another easy instrument for the primary care clinician to use is the LIE/BET questionnaire [12]. This tool has been validated in adult and adolescent populations [13]. It is composed of two questions selected from the DSM-IV criteria for pathological gambling because they were identified as the best predictors of pathological gambling. A yes answer to either question suggests the need to investigate for the presence of additional criteria for disordered gambling.

In Western settings, the estimated lifetime prevalence of gambling disorder is less than 2% [14]. Although the majority of adults who gamble do so on a social basis and do not experience long-term or permanent problems, this type of gambling behavior, known as social gambling, is different than disordered gambling [15]: Mortality and suicide rates are significantly elevated among pathological gamblers, and gambling disorder seems to be an independent risk factor for suicide [16].

Data regarding the role played by demographic factors in gambling disorder is inconsistent. Nonetheless, a number of demographic groups have a higher rate of gambling problems than the general population. These factors include males, young age, adults being treated for a psychiatric problem, substance abuse, the presence of a family history of gambling disorder and a low-socioeconomic status [17].

The main pathological comorbidities of gambling disorder are substance addictions (including tobacco, alcohol), mood disorders (including depression) and anxiety disorders (including generalized anxiety disorder) [18]. The coexistence of alcohol use disorder and disordered gambling is prevalent among young adults and may increase the negative repercussions associated with each disorder [19]. Daily cigarette smoking in pathological gamblers is also very frequent and is associated with a more severe gambling behavior and more financial problems [20]. Furthermore, trait impulsivity in adolescents and adults is associated with probable pathological gambling and use of tobacco-related products, including water pipe smoking (WPS) [21]. Gambling disorder is also associated with the presence of depressive symptoms, with the possibility of sharing some common etiological factors [22]. Moreover, probable pathological gambling is positively associated with the development of generalized anxiety disorder (GAD) [23].

In Lebanon, especially since the economic crisis and the COVID-19 pandemic, there is an increase in the prevalence of mental health disorders, including stress, depression and anxiety [24]. The COVID-19 pandemic and the lockdown seem to not only increase gambling behavior in pathological gamblers, but also contribute to the occurrence of new cases of gambling disorder [25]. The prevalence of gambling disorder in Lebanese university students is high [26]. The South Oaks Gambling Screen (SOGS), the most used tool in the world in terms of pathological gambling, has never been translated to Arabic, nor culturally adapted in a way that suits the Lebanese population and validated. This study’s objectives were to validate an Arabic-language version of the SOGS and assess factors associated with probable pathological gambling among a sample of Lebanese adults.

## Methods

### Study design

This study was carried out between February and April 2021, amid lockdown due to the COVID-19 pandemic. A total of 601 individuals participated in this study by filling the online questionnaire. The sample was selected based on different demographic backgrounds from the five governorates of Lebanon (Beirut, Bekaa, Mount Lebanon, South Lebanon and North Lebanon) using a snowball technique. We contacted friends and family members to fill the online survey, then each participant was asked to forward the survey link to other friends and family members. Respondents were briefed about the topic, and the different aspects of the questionnaire before filling it out, while being assured of the confidentiality of their answers by one of the collaborators of this research project. Inclusion criteria included people above 18 years old living in Lebanon.

### Minimal sample size calculation

According to the G-power software, and based on an effect size f2 = 2%, an alpha error of 5%, a power of 80%, and taking into consideration 20 factors to be entered in the multivariate analysis, the results showed that a minimal number of 395 was needed.

### Questionnaire

The questionnaire sent to the participants was in the Arabic language, which is the official language of Lebanon as well as the native language of all respondents. The questionnaire includes a sociodemographic section, and a scale-based section regarding different factors, as follows:

#### Sociodemographic data

In this part of the questionnaire, participants were asked about their general sociodemographic data, including their age, gender, educational level, income, marital status, number of children, physical activity index and information about the household crowding index of the participants. The physical activity index was obtained by multiplying the daily activity intensity, frequency and duration [27],whereas the household crowding index was calculated by dividing the number of persons living in the house by the number of rooms excluding the kitchen and bathrooms.

#### Scale-based section

The following scales were used in the questionnaire:

- *Arabic Version of the SOGS (South Oaks Gambling Screen)*

This is a questionnaire consisting of 20 rated items, at a rate of 1 point per item. The SOGS scores are interpreted as follows: A score of 0 to 2 indicates no problem with gambling, a score of 3 or 4 indicates some problem with gambling, and a score of 5 or more indicates probable pathological gambling. The cutoff score of 5 has been discussed, especially in some countries like Australia, after very high prevalence rates (> 6%) were found using this cutoff. Thus, some studies propose a cut-off score of 10 making it possible to obtain prevalence rates similar to those found in the international literature. The sensitivity of this questionnaire is estimated at 99%. Its specificity is estimated at 83%, which results in a risk of false positives [28]. (Cronbach’s alpha in this study = 0.947) The permission to use this questionnaire was obtained from Dr. Henry Lesieur and the South Oaks Foundation. The original SOGS was used with a lifetime-based measure.

- *Alcohol Use Disorders Identification Test*

The Alcohol Use Disorders Identification Test (AUDIT) was developed by the World Health Organization in 1982 as a simple way to assess alcohol consumption, drinking behaviors, and alcohol-related problems [29]. It is a 10-item screening tool used to detect risky and harmful drinking patterns during the past 12 months. The answers are scored on a point system; a score of 8 or more is the threshold for identifying hazardous or harmful alcohol consumption [29]. (Cronbach’s alpha in this study = 0.857).

- *Fagerström Nicotine Dependence Test*

The Fagerström Test for Nicotine Dependence, validated in Lebanon [30], is a standard instrument for assessing the intensity of physical addiction to nicotine. This test was designed to provide an ordinal measure of nicotine dependence related to cigarette smoking. It contains six items that evaluate the quantity of cigarette consumption, the compulsion to use, and dependence. In the scoring of the Fagerström Test for Nicotine Dependence, yes/no items are scored 0 or 1 and multiple-choice items are scored from 0 to 3. The items are summed to yield a total score of 0–10. The higher the total Fagerström score, the more intense is the patient's physical dependence to nicotine [31]. (Cronbach’s alpha in this study = 0.628).

- *Lebanese Waterpipe Dependence Scale*

The Lebanese Waterpipe Dependence Scale-11 (LWDS-11) was used to assess waterpipe dependence [32]. The LWDS-11 is composed of 11 items, measured on a 4-point Likert scale ranging from 0 to 3, and four subscales, which are described as physiological nicotine dependence (items 1–4), termination of dysphoric states or negative reinforcement (items 5 and 6), psychological craving (items 7–9), and positive reinforcement (items 10 and 11). The total score is reached by adding up the corresponding points and indicates the level of dependence. The LWDS-11 discriminated between mild, moderate, and heavy waterpipe smokers, based on a threshold score of 10 [33]. (Cronbach’s alpha for the total scale in this study = 0.777).

- *GAD-7*

This is an easy-to-use self-administered patient questionnaire used as a screening tool and severity measure for generalized anxiety disorder (GAD) [34]. GAD-7 has seven items: (1) nervousness; (2) inability to stop worrying; (3) excessive worry; (4) restlessness; (5) difficulty in relaxing; (6) easy irritation; and (7) fear of something awful happening. The GAD-7 score is calculated by assigning scores of 0, 1, 2, and 3, to the response categories of 'not at all', 'several days', 'more than half the days', and 'nearly every day', respectively, and adding together the scores of the seven questions. Scores of 5, 10, and 15 are taken as the cut-off points for mild, moderate and severe GAD, respectively. When used as a screening tool, further evaluation is recommended when the score is 10 or greater. (Cronbach’s alpha for the total scale in this study = 0.939) [35]. The GAD-7 is validated in the Arabic language in Lebanon [36].

- *PHQ-9*

The PHQ-9 (Patient Health Questionnaire-9) is a 9-question instrument given to patients in a primary care setting to screen for the presence and severity of depression according to DSM-4 criteria. This questionnaire takes less than 3 min to complete. Scores range from 0 to 27. In general, a total of 10 or above is suggestive of the presence of depression [37]. (Cronbach’s alpha for the total scale in this study = 0.926). The PHQ-9 is validated in the Arabic language in Lebanon [36].

### Translation procedure of the SOGS

Two independent certified translators performed the forward translation (from English to Arabic), and adapted it in a way that suits the Lebanese culture. Then, they reviewed, reconciled and harmonized this translation. The back-translation (From Arabic to English) was performed by two other independent certified translators, who were unaware of the intended concepts the questionnaire measures and were very fluent in English. Then, a committee of experts including healthcare professionals and both the forward and backward translators matched the back-translation with the original English questionnaire to detect inconsistencies and solve discrepancies between the two versions, and reached a consensus on all items to produce a pre-final version of the forward translation. This pre-final version was pilot tested on a small convenience sample of 12 people, to make sure that the translated questions retained the same meaning as the original questions, and ensure that there was no confusion regarding the translated items. The product of this process was the finalized forward translation.

### Statistical analysis

The total sample was divided into two subsamples; sample 1 (n = 401) served to perform the factor analysis (FA), whereas sample 2 (n = 200) served for the confirmatory factor analysis. The FACTOR software was used to perform the FA, using the polychoric (tetrachoric) correlation and using the parallel analysis as a procedure for determining the number of factors/components. The varimax rotation was used to extract the items since the latter were not highly correlated. The Kaiser–Meyer–Olkin (KMO) and the Bartlett’s test of sphericity p-value were calculated to ensure model’s adequacy. Similarly, a confirmatory factor analysis was performed using the same software; the GFI and AGFI values were calculated (both are Chi-square-based calculations independent of degrees of freedom), with values ≥ 0.90 considered acceptable [38]. In addition, the root mean square error of approximation (RMSEA) and the comparative fit index (CFI) were used as these are the most commonly used indices [39]. Values of RMSEA of ≤ 0.06 indicate a good-fitting model, while CFI values > 0.90 indicate a reasonably good fit of the model [38]. The SPSS software v.25 was used for the remaining statistical analysis. The Kuder-Richardson 20 (KR20; equivalent to the Cronbach’s alpha value) value was calculated to indicate the internal reliability of the SOGS scales’ items. Since the SOGS total score did not follow a normal distribution, as verified by the skewness and kurtosis values (outside the range of − 2 and + 2), we divided the total score into 3 categories as follows: no gambling problems (scores between 0 and 2), some gambling problems (scores 3 or 4) and probable pathological gambling (scores of 5 or more). The Chi-square and ANOVA tests were used to evaluate the associations between the SOGS categories and categorical variables and continuous variables respectively. A multinomial regression was conducted taking the SOGS categories as the dependent variable, to assess factors associated with having some gambling problems and probable pathological gambling compared to no gambling problems (taken as the reference group). Variables *p* < 0.2 in the bivariate analysis were taken as independent ones in the multivariate analysis model. Significance was set at *p* < 0.05.

## Results

### Sociodemographic and other participants’ characteristics

A total of 601 accepted to participate in this study. The mean age of the participants was 24.89 ± 11.11 years, with 65.4% females. The results also showed that 16 (2.7%) of the participants had some gambling problems, whereas 39 (6.5%) of them had probable pathological gambling. Other details are summarized in Table 1.

**Table 1:** Sociodemographic and other participants’ characteristics (N = 601)

Variable	N (%)
*Gender*	
Male	208 (34.6%)
Female	393 (65.4%)
*Marital status*	
Single	476 (79.2%)
Married	125 (20.8%)
*Education*	
Complementary or less	26 (4.3%)
Secondary	62 (10.3%)
University	513 (85.4%)
*SOGS categories*	
No gambling problems	546 (90.8%)
Some gambling problems	16 (2.7%)
Probable pathological gambling	39 (6.5%)
Waterpipe dependence (yes; ≥ 10)	71 (11.8%)
Alcohol use disorder (yes; ≥ 8)	12 (2.0%)
*FTND categories*	
Very low (0–2)	552 (91.8%)
Low (3–4)	22 (3.7%)
Moderate (5)	6 (1.0%)
High (6–7)	15 (2.5%)
Very high (8–10)	6 (1.0%)
*GAD-7 categories*	
None (0–5)	280 (46.6%)
Low (6–10)	154 (25.6%)
Moderate (11–15)	99 (16.5%)
Severe (16 and above)	68 (11.3%)
*PHQ-9 categories*	
None	235 (39.1%)
Mild	154 (25.6%)
Moderate	103 (17.1%)
Moderately severe	58 (9.7%)
Severe	51 (8.5%)

	Mean ± SD
Age (in years)	24.89 ± 11.11
Number of children	0.59 ± 1.38
Physical activity index	25.69 ± 20.94
Household crowding index	1.09 ± 0.57
Cigarette dependence	0.49 ± 1.53
Waterpipe dependence	2.45 ± 5.20
Problematic alcohol use	0.54 ± 2.31
Generalized anxiety disorder	7.32 ± 6.03
Depression	8.27 ± 7.03

#### Factor analysis

A factor analysis, using the principal component analysis, was performed on the SOGS scale items. All items remained and were divided into three factors (Factor 1 “Loss of control”; Factor 2 “Source of funds for gambling”; Factor 3 “Financial, psychological and social consequences of gambling”). The KMO value = 0.881 and the Bartlett test of sphericity *p* < 0.001 ensured the model adequacy. The SOGS items were able to explain 73.35% of the variance, with an internal reliability of KR20 = 0.947 for the total scale (Table 2).

**Table 2:** Factor analysis of the South Oaks Gambling Scale items using the varimax rotation

Variable	Factor 1	Factor 2	Factor 3
1. When you gamble, how often do you go back another day to win back money you lost?	0.362		
2 Have you ever claimed that you won money gambling but you had in fact lost?	0.871		
3. Did you ever feel that you have had problem with betting money or gambling?	0.822		
4. Did you ever gamble more than you intended to?			0.880
5. Have you received criticism because of your gambling problem, regardless of whether or not you thought it was true?			0.690
6. Have you ever felt guilty about the way you gamble or what happens when you gamble?			0.864
7. Have you ever felt like you would like to stop betting or gambling but didn't think you could?			0.714
8. Have you ever hidden betting slips, lottery tickets, gambling money, an “I owe you” or other signs of betting or gambling from your partner, children or other important people in your life?			0.897
9. Have money arguments ever centered on your gambling problem?			0.889
10. Have you ever borrowed money from someone and did not pay it back as a result of your gambling?			0.718
11. Did you ever not go to work or school due to betting money or gambling?			0.918
12. If you borrowed money to gamble or to pay gambling debts, from whom or where did you borrow such amount?- from household money		0.752	
13. If you borrowed money to gamble or to pay gambling debts, from whom or where did you borrow such amount?- from your husband/wife or partner		0.627	
14. If you borrowed money to gamble or to pay gambling debts, from whom or where did you borrow such amount?- from other relatives or in-laws		0.665	
15. If you borrowed money to gamble or to pay gambling debts, from whom or where did you borrow such amount?—from banks, loan companies or credit unions		0.806	
16. If you borrowed money to gamble or to pay gambling debts, from whom or where did you borrow such amount?- from credit cards		0.683	
17. If you borrowed money to gamble or to pay gambling debts, from whom or where did you borrow such amount?—from loan sharks		0.751	
18. If you borrowed money to gamble or to pay gambling debts, from whom or where did you borrow such amount?- you cashed in stocks, bonds or other securities		0.666	
19. If you borrowed money to gamble or to pay gambling debts, from whom or where did you borrow such amount?- you sold personal or family properties		0.737	
20. If you borrowed money to gamble or to pay gambling debts, from whom or where did you borrow such amount?- you borrowed money or gave rubber checks		0.650	
Percentage of variance explained	54.53	10.85	7.97
Cronbach’s alpha	0.691	0.929	0.954

Factor 1: “Loss of control”

Factor 2: “Source of funds for gambling”

Factor 3: “Financial, psychological and social consequences of gambling”

#### Confirmatory factor analysis

The results of the CFA confirmed the results of the EFA as follows: RMSEA = 0.033, CFI = 0.996, Goodness of Fit Index (GFI) = 0.982 and Adjusted Goodness of Fit Index (AGFI) = 0.975.

#### Bivariate analysis

A significantly higher percentage of participants with probable pathological gambling were males (compared to females) and had a secondary level of education compared to the other categories. Moreover, participants with probable pathological gambling had more cigarette dependence, generalized anxiety disorder and depression compared to those with no gambling problems or some gambling problems. Finally, participants who had some gambling problems had more waterpipe dependence and problematic alcohol use compared to the other two categories (Table 3).

**Table 3:** Bivariate analysis of factors associated with the gambling categories

Variable	SOGS categories			*p*
	No gambling problems (N = 546)	Some gambling problems (N = 16)	Probable pathological gambling (N = 39)	
*Gender*				**< 0.001**
Male	176 (84.6%)	7 (3.4%)	25 (12.0%)	
Female	370 (94.1%)	9 (2.3%)	14 (3.6%)	
*Marital status*				0.398
Single	430 (90.3%)	15 (3.2%)	31 (6.5%)	
Married	116 (92.8%)	1 (0.8%)	8 (6.4%)	
*Education*				**0.01**
Complementary or less	26 (100.0%)	0 (0%)	0 (0%)	
Secondary	50 (80.6%)	6 (9.7%)	6 (9.7%)	
University	470 (91.6%)	10 (1.9%)	33 (6.4%)	
Age (in years)	25.17 ± 11.43	23.94 ± 8.85	21.39 ± 5.54	0.220
Number of children	0.62 ± 1.42	0.50 ± 1.10	0.26 ± 0.79	0.420
Physical activity index	25.77 ± 21.18	21.38 ± 15.97	26.36 ± 19.52	0.696
Household crowding index	1.06 ± 0.50	1.25 ± 0.55	1.31 ± 1.15	0.394
Cigarette dependence	0.44 ± 1.50	0.56 ± 1.79	1.10 ± 1.86	**0.007**
Waterpipe dependence	2.20 ± 4.80	5.50 ± 7.05	4.61 ± 8.20	**0.019**
Problematic alcohol use	0.36 ± 1.53	2.31 ± 4.47	2.26 ± 6.17	**0.013**
Generalized Anxiety Disorder	6.96 ± 5.95	10.44 ± 6.08	11.18 ± 5.57	**< 0.001**
Depression	7.73 ± 6.79	12.13 ± 6.17	14.23 ± 7.61	**< 0.001**

Numbers in bold indicate significant *p* values

#### Multivariate analysis

A multinomial regression was conducted taking the SOGS categories as the dependent variable, to assess factors associated with having some gambling problems and probable pathological gambling compared to no gambling problems (taken as the reference group). The results of the first model showed that more problematic alcohol use (higher AUDIT scores) (aOR = 1.19) was significantly associated with higher odds of having some gambling problems (Table 4, Model 1). The results of the second model showed that more problematic alcohol use (higher AUDIT scores) (aOR = 1.17), and more depression (aOR = 1.13) were significantly associated with higher odds of probable pathological gambling, whereas females (aOR = 0.27) had significantly lower odds of probable pathological gambling compared to males (Table 4, Model 2).

**Table 4:** Multivariate analysis: Multinomial regression taking the SOGS categories as the dependent variable

Variable	*p*	aOR	95% CI
Model 1: Having some gambling problems vs no gambling problems*			
Problematic alcohol use	0.006	1.19	1.05–1.34
Model 2: Probable pathological gambling vs no gambling problems*			
Problematic alcohol use	0.002	1.17	1.06–1.29
Depression	0.002	1.13	1.05–1.21
Gender (females vs males*)	0.001	0.27	0.12–0.57

*Reference group; aOR = adjusted odds ratio; CI = Confidence Interval; variables entered in the model: cigarette dependence, waterpipe dependence, problematic alcohol use, generalized anxiety disorder, depression, education, gender; Nagelkerke pseudo R^2^ = 20.2%

## Discussion

This study, the first of its kind in Lebanon, was able to validate the SOGS scale in Arabic and assess certain factors **(**problematic alcohol use, depression and gender) associated with probable pathological gambling among a sample of Lebanese adults.

### Scale validation

The Arabic version of the South Oaks Gambling Screen (SOGS) items demonstrated excellent internal consistency and reliability with a high KR-20 coefficient = 0.947 (> 0.9). In our study, the factor analysis of the scale's structure resulted in a three-dimensional solution. From the three evidenced factors, factor two was mainly composed by questions related to the source of funds for gambling, whereas factor three was related mainly to the financial, psychological and social consequences of gambling. Factor one, composed only by three questions, included items assessing “chasing”, lying about one’s gambling, and feeling that one had a gambling problem. This factor is analogous to the underlying construct of “loss of control” over one’s gambling which gambling researchers now distinguish from “harms” [40].

Comparing our results with other SOGS validation studies, the internal consistency value was higher than the values reported for the original version [8], the Chinese version [41] and the Brazilian version [42]. In fact, for the Chinese version of the SOGS, the Cronbach’s alpha value of the 18-item SOGS scale after deleting two items with zero endorsement was 0.84, and in the general sample, the principal component analysis indicated, unlike our study, one primary factor [41]. For the Brazilian version of the SOGS, the internal consistency measured by Cronbach's alpha was 0.9304. Factor analysis resulted in a three-dimensional solution like our study, but the questions were grouped differently [42]. When the Reliability and validity of the English version of the South Oaks Gambling Screen were evaluated in a multiracial Asian community sample in Singapore, the SOGS demonstrated high internal consistency with Cronbach's alpha coefficient of 0.84, and CFA supported a one-factor solution (unlike our study) for the SOGS [43].

Those results were confirmed when performing the confirmatory factor analysis, demonstrating that the Arabic version of the SOGS scale, according to the initial psychometric properties, might be a valid scale for the assessment of gambling among Lebanese adults. Further studies are to be conducted among a random sample of the general population to confirm our findings.

### Gambling problems rate

The rate of probable pathological gambling in our sample was 6.5%. This number is high compared to global prevalence figures [44]. We have to remember that the specificity of the South Oaks Gambling Screen (SOGS) is estimated at 83% [28], which means there is a risk of false positives, especially that the original SOGS with a lifetime-based measure was used. Furthermore, the sampling method may have resulted in the high rate of gambling problems in our sample due to gamblers possibly recruiting other gamblers via snowball sampling or chain-referral sampling. This could also be explained by the fact that Lebanon is a country in the Middle-East where gambling is legal. Many types of games are available in Lebanon at: Casino du Liban, amusement centers, and horse racing tracks. In addition, the relatively recent emergence of online gambling should also be noted. Casino du Liban is a major tourist destination in the region and is constantly vibrating with traditional and international cultural activities, entertainment programs, culinary events, beauty contests, poker tournaments and much more. It has become the favorite destination for gamers, theatre lovers, art lovers, music lovers and epicureans. Could the relatively easy access to this casino and the beautiful image it displays have an impact on the higher rate of pathological gamblers found in our sample? This remains to be determined.

Furthermore, the current poor socioeconomic situation in Lebanon these days could also explain the higher rate of pathological gamblers in our sample; low-income citizens may resort to gambling to earn more money. The economic crisis alongside the COVID-19 pandemic are also causing more stress and depression among Lebanese citizens [24], and one of the DSM-5 criteria for the diagnosis of “gambling disorder” is: “Often gambles when feeling distressed (e.g., helpless, guilty, anxious, depressed)” [1].

### Correlates of gambling problems

Male gender was found to be associated with more gambling disorder, in line with previous findings [45]. This could be because men tend to take more risks than women; men are also much more “high sensation seekers” than women [46].

Furthermore, more problematic alcohol use was significantly associated with the presence of some gambling problems and probable pathological gambling. This is also in agreement with previous studies [18, 23, 44, 47, 48] and could be explained by the presence of comorbidities between substance addiction (tobacco, alcohol) and behavioral addiction (gambling disorder) [18]. In fact, substance-related disorders (including alcohol use disorder) and disordered gambling are frequently associated, and preventing substance abuse may reduce gambling disorder rates [49]. The coexistence of alcohol use disorder and probable pathological gambling is frequent among young adults and may increase the negative consequences associated with each disorder [19].

Moreover, more depression was significantly associated with more probable pathological gambling, in agreement with former studies [18, 22, 49, 50]. In fact, in a meta-analysis of 11 studies, apart from substance addictions and anxiety disorders, mood disorders were the main comorbidity of gambling disorder [18]. Mood disorders are frequently seen in pathological gamblers with comorbidity rates as high as 75 percent for unipolar depression [51]. Probable pathological gambling is associated with the presence of depressive symptoms, with the possibility of sharing common etiological factors [22].

The findings of our study show the association between gambling disorder and other mental health disorders. These findings may justify the need to screen for gambling disorder in the context of other psychiatric disorders, especially in Lebanon where there is now a reliable and valid Arabic-language version of the SOGS suitable for the Lebanese context.

### Limitations

Our study being a cross sectional study, a limitation would be that probable pathological gambling and its associated factors are simultaneously assessed, so there would be no evidence of a temporal relationship between them. Furthermore, an information bias could have arisen in the data collection. A selection bias could also be found since convenience sampling that did not include random selection of participants, was adopted in our study that was conducted amid lockdown due to the COVID-19 pandemic (snowball sampling or chain-referral sampling); consequently, the results of this study cannot be generalized to the whole population. A residual confounding bias may also be present because there were additional confounding factors that may have been associated with probable pathological gambling that were not considered and there was no attempt to adjust for them, because data on these factors was not collected. Moreover, the south oaks gambling screen (SOGS) overestimates pathological gambling when applied in a general population context [6, 8], especially that the original SOGS with a lifetime time frame was used in our study, which has been criticized for failing to discriminate between current cases and those in remission and overestimating the rate of probable pathological gamblers. More psychometric properties of the SOGS scale are needed in future studies (test–retest, convergent validity with other scales known to measure gambling disorder such as the Canadian Problem Gambling Index (CPGI), etc. All scales that were used in the questionnaire were in Arabic. However, the AUDIT scale has not yet been validated in the Arabic language in Lebanon. Finally, the percentage of participants with problematic alcohol use, cigarette and waterpipe dependence was low; therefore, results should be interpreted with caution. Future studies, taking all these limitations into consideration, are recommended.

## Conclusion

Our study has validated an Arabic-language version of the South Oaks Gambling Screen (SOGS) for use in Lebanon, and showed some factors associated with probable pathological gambling (male gender, alcohol use disorder and depression). We hope that this reliable and valid version contributes towards better screening for gambling disorder in Lebanon. These results may serve as a first step to raise the collective consciousness about the depth of this problem in order to identify patients who are problem or pathological gamblers, and encourage the implementation of primary prevention of this disease through educational programs targeting young people and adults at risk, which could contribute to reducing gambling problems and establishing a culture of controlled and responsible gambling behaviors, especially that gambling is becoming more and more accessible with the rise of online gambling.

## Data Availability

The datasets generated and/or analysed during the current study are not publicly available as per their institutions’ policies but are available from the corresponding author on reasonable request.
